# Histone deacetylase inhibitors dysregulate DNA repair proteins and antagonize metastasis-associated processes

**DOI:** 10.1007/s00432-019-03118-4

**Published:** 2020-01-13

**Authors:** Nicole Kiweler, Désirée Wünsch, Matthias Wirth, Nisintha Mahendrarajah, Günter Schneider, Roland H. Stauber, Walburgis Brenner, Falk Butter, Oliver H. Krämer

**Affiliations:** 1 Department of Toxicology, University Medical Center of the Johannes Gutenberg University Mainz, Obere Zahlbacher Straße 67, 55131 Mainz, Germany; 2 Department of Otorhinolaryngology, University Medical Center Mainz, 55131 Mainz, Germany; 3 Klinik Und Poliklinik für Innere Medizin II, Technical University of Munich, 81675 Munich, Germany; 4 Clinic for Obstetrics and Women’s Health, University Medical Center Mainz, 55131 Mainz, Germany; 5 Department of Urology, University Medical Center Mainz, 55131 Mainz, Germany; 6 Institute of Molecular Biology (IMB), 55128 Mainz, Germany; 7 Present Address: Department of Oncology, Luxembourg Institute of Health, 1445 Strassen, Luxembourg; 8 Present Address: Hematology and Oncology, Charité-Universitätsmedizin Campus Benjamin Franklin, 12200 Berlin, Germany

**Keywords:** Adhesion, EMT, DNA damage, HDACi, MET, TGFβ

## Abstract

**Purpose:**

We set out to determine whether clinically tested epigenetic drugs against class I histone deacetylases (HDACs) affect hallmarks of the metastatic process.

**Methods:**

We treated permanent and primary renal, lung, and breast cancer cells with the class I histone deacetylase inhibitors (HDACi) entinostat (MS-275) and valproic acid (VPA), the replicative stress inducer hydroxyurea (HU), the DNA-damaging agent cis-platinum (L-OHP), and the cytokine transforming growth factor-β (TGFβ). We used proteomics, quantitative PCR, immunoblot, single cell DNA damage assays, and flow cytometry to analyze cell fate after drug exposure.

**Results:**

We show that HDACi interfere with DNA repair protein expression and trigger DNA damage and apoptosis alone and in combination with established chemotherapeutics. Furthermore, HDACi disrupt the balance of cell adhesion protein expression and abrogate TGFβ-induced cellular plasticity of transformed cells.

**Conclusion:**

HDACi suppress the epithelial–mesenchymal transition (EMT) and compromise the DNA integrity of cancer cells. These data encourage further testing of HDACi against tumor cells.

**Electronic supplementary material:**

The online version of this article (10.1007/s00432-019-03118-4) contains supplementary material, which is available to authorized users.

## Introduction

The formation of metastases that originate from a primary cancer is commonly associated with increased drug resistance and patient death (Fidler and Kripke 2015). EMT and the subsequent transition of cells back to the mesenchymal state have been associated with metastasis for decades (Nieto 2013; Zeisberg and Neilson 2009). However, there is an ongoing dispute whether EMT is a prime event in the metastatic process or whether the mesenchymal phenotype of breast and pancreatic cancer cells represents predominantly an indicator of cellular resistance to DNA damage (Aiello et al. 2017; Brabletz et al. 2018; Fischer et al. 2015; Ye et al. 2017; Zheng et al. 2015). Irrespective of this conceptual conflict, it is undoubted that novel drugs are necessary to combat clinical metastasis formation to enhance patient survival. Such drugs should be analyzed for their impact on both genomic integrity and modulation of EMT.

HDACi are epigenetic drugs that enhance protein acetylation and thereby impact a large number of cellular functions (Bayat Mokhtari et al. 2017; Mrakovcic et al. 2019; Müller and Krämer 2010; Nikolova et al. 2017; Vancurova et al. 2018). Since the Food and Drug Administration has approved four HDACi for the treatment of hematological malignancies, additional research is warranted to demonstrate how a pharmacological inactivation of HDACs affects metastasis formation. To solve this issue, global assays and analyses of various tumor cell types are required. We recently revealed that HDACi did not shift renal cell carcinoma (RCC) cells to a distinct epithelial or mesenchymal phenotype, but rather disrupted functional EMT/MET protein expression signatures and triggered apoptosis of RCC cells (Kiweler et al. 2018). These data are coherent with previous reports that show beneficial effects of HDACi against RCC cells and EMT (Chun 2018; Jones et al. 2009a, b; Juengel et al. 2014; Mao et al. 2017). Furthermore, HDACi counteracted the acquired resistance of RCC cells against the mammalian target of rapamycin-inhibitor everolimus and the glucose-regulating biguanide metformin (Juengel et al. 2014; Wei et al. 2018). In light of the chemoresistance and the poor prognosis of metastatic RCC (Barbieri et al. 2017; Chang et al. 2019), these findings suggest that HDACi pose an interesting therapeutic option for this cancer type.

The analysis of drug-dependent effects on metastasis should also involve conditions that promote this process. The secreted cytokine TGFβ is a tumor suppressor in non-transformed cells through its cell cycle arresting activity. Tumor cells, including those from RCC, are insensitive to this effect of TGFβ and undergo metastasis-promoting EMT and acquire chemotherapy resistance (Hao et al. 2019; Singla et al. 2018; Tretbar et al. 2019). These processes can be abrogated with HDACi in RCC cells. The HDACi trichostatin-A (TSA) and butyrate suppressed TGFβ-induced EMT (Yoshikawa et al. 2007) and VPA prevented the TGFβ-dependent activation of the EMT-associated transcription factor SMAD4 (Mao et al. 2017). Such effects could be particularly relevant to tumors that are or become resistant to standard cancer drugs such as renal and lung tumors (Barbieri et al. 2017; Chang et al. 2019; Dietrich and Gerber 2016; Foy et al. 2017).

In light of the recent discussion on the impact of DNA repair and EMT for the metastatic process and its relation to acquired chemoresistance, and due to the influence of HDACi on the EMT of RCC cells, we set out to investigate if HDACi affect the expression of DNA repair proteins, including p53, and cell adhesion molecules. In addition, we investigated whether HDACi modulate TGFβ-induced cell plasticity and if the combination of HDACi and L-OHP or HU is effective against cancer cells.

## Results

### HDACi suppress DNA repair protein expression

**Fig. 1:**
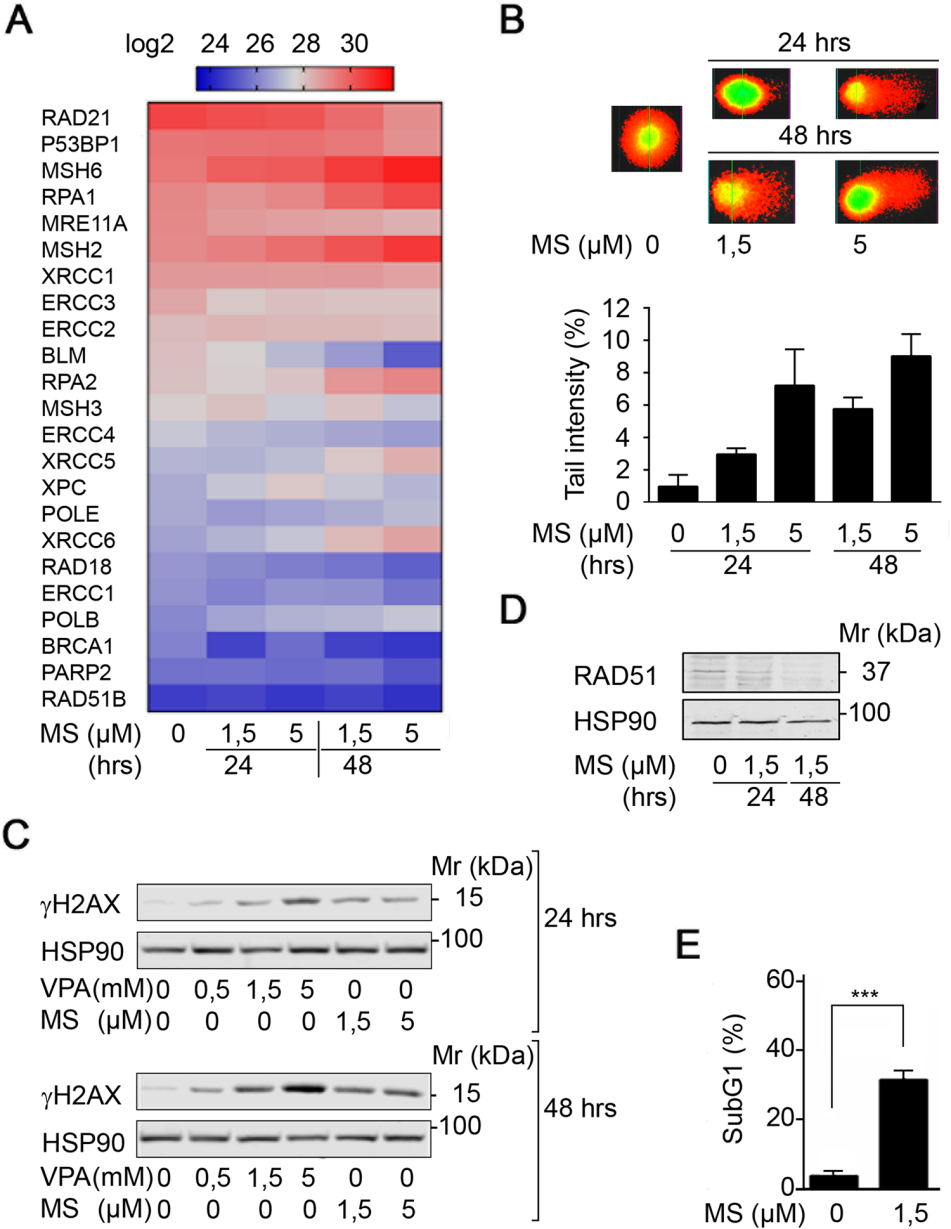
HDACi trigger replication stress and DNA damage. **a** Renca cells were treated with the indicated concentrations of MS-275 (μM) for 24 to 48 h. Four independent replicates were analyzed for protein expression via LFQ in mass spectrometry. Heatmap depicts changes in LFQ expression levels of proteins with implications for the maintenance of genomic integrity. Statistics regarding changes in protein expression are provided in Supplementary Fig. S1. Several of the proteins are important for various DNA repair pathways and pathway choice. Proteins mainly relevant to DNA repair and HR are RAD21, 53BP1, RPA1, MRE11A, RPA2, XRCC5, POLE, XRCC6, RAD18, POLB, BRCA1, PARP2, RAD51B; critical for MMR are MSH6, MSH2, MSH3; for BER is XRCC1; for NER are ERCC3, ERCC2, BLM, ERCC4, XPC, ERCC1. **b** Renca cells were treated with the indicated concentrations of MS-275 (μM) for 24 h and 48 h. Tail intensity was quantified for *n* = 2 independent experiments. **c** Renca cells were treated with the indicated concentrations of MS-275 (μM) and VPA (mM) for 24 h and 48 h, and analyzed for phosphorylation of H2AX (Ser139) by Western blot. HSP90 serves as loading control; Mr (kDa), relative molecular mass in kilo Daltons (kDa). **d** Mz-ccRCC2 cells were treated with 1.5 μM MS-275 for 24 and 48 h. RAD51 expression was analyzed on Western blot. HSP90 served as loading control; Mr (kDa), relative molecular mass in kilo Daltons (kDa). **e** Mz-ccRCC2 cells were treated for 48 h with 1.5 μM MS-275 and cell death was determined as percentage of subG1 population in fixed, PI-stained cells using flow cytometry. Graph shows mean ± SD (*n* = 5); two-tailed unpaired *t* test with Welch’s correction, ***p value < 0.001

RCC-derived Renca cells are sensitive to pro-apoptotic effects of class I HDACi (Kiweler et al. 2018). Due to the rising interest in the impact of HDACs and HDACi on DNA replication and DNA integrity, we analyzed the expression levels of proteins that control replicative stress and DNA damage in a previously published proteome database for MS-275-treated Renca cells (Kiweler et al. 2018). This analysis showed that HDAC inhibition by MS-275 decreased the expression of proteins that control homologous recombination (HR), DNA mismatch repair (MMR), nucleotide excision repair (NER), and base excision repair (BER) in Renca cells (Fig. 1a and Supplementary Fig. S1).

To assess if the observed alterations in protein expression result in DNA damage at a global level, we used the comet assay technique, which is also known as single cell electrophoresis assay. MS-275 evoked a time- and dose-dependent increase in DNA tails indicating DNA damage (Fig. 1b).

Due to these findings, we analyzed the phosphorylation of histone H2AX at serine-139 (ɣH2AX), which is a marker for replication stress, single-/double-strand breaks in DNA, and apoptosis (Nikolova et al. 2017; Rogakou et al. 1998). MS-275 and VPA, which we used as additional class I HDACi (Bradner et al. 2010; Müller and Krämer 2010), induced a time- and dose-dependent accumulation of ɣH2AX (Fig. 1c).

These data encouraged us to test for a dysregulation of DNA repair factors and DNA injury upon class I HDAC inhibition in primary human RCC cells (Mz-ccRCC2). We found that MS-275 reduced the expression of the recombinase RAD51, which is a key factor for HR-mediated DNA repair (Nikolova et al. 2017), in primary RCC cells (Fig. 1d). This was associated with a highly significantly increased level of DNA fragmentation, which is typically seen in cells undergoing apoptosis (Fig. 1e).

We conclude that the activity of class I HDACs is necessary for the expression of proteins that control genomic integrity. Accordingly, HDACi cause replicative stress and DNA damage in RCC cells.

### Status of p53 and its regulation by HDACi in Renca cells

The tumor suppressor p53 determines the fate of HDACi-treated cells (Mrakovcic et al. 2019) and is relevant for DNA replication and HR (Gottifredi and Wiesmüller 2018; Klusmann et al. 2016). As we see replication stress/DNA damage in HDACi-treated cells (Fig. 1), we consequently analyzed the p53 status of and a putative regulation of p53 expression by HDACi in Renca cells. We searched major databases for the characterization of cell lines concerning the p53 status of the Renca cell line. One database provided information that Renca cells carry one R210C exchange in the DNA-binding domain of p53 (Zeitouni et al. 2017). Meta-data analysis of whole-exome sequencing data (WES data; accession no. PRJEB12925) for Renca cells from Mosely et al. (2017) revealed that these cells have one wild-type p53 allele and one allele with the R210C mutation (Supplementary Fig. S2).

**Fig. 2:**
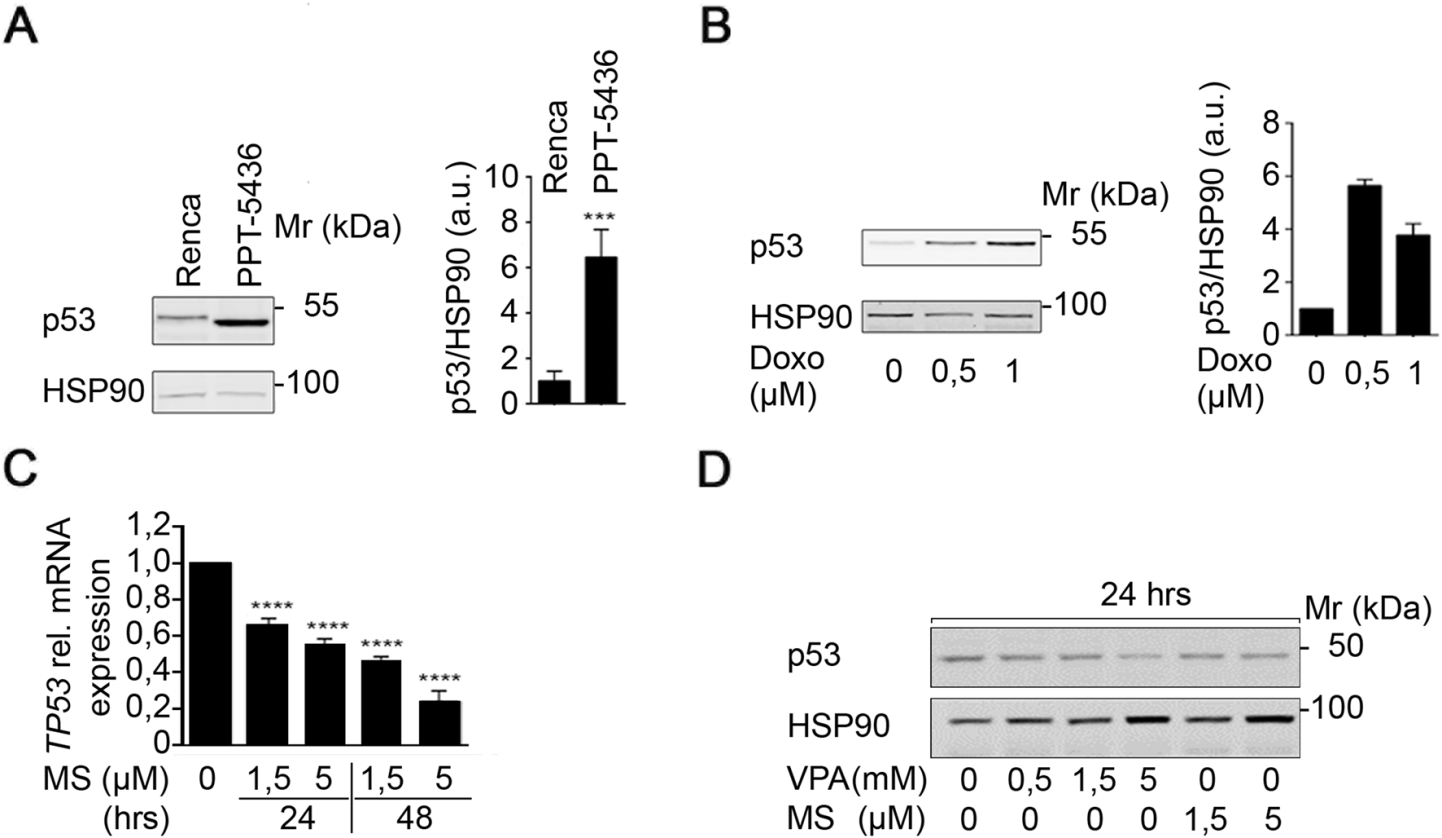
Status of p53 and regulation through HDACi in Renca cells. **a** Untreated Renca and PPT-5436 cells were analyzed for p53 expression on Western blot. HSP90 served as loading control. Quantification of signal intensities was accomplished by densitometric analysis and subsequent normalization to the respective loading controls. Graph displays mean ± SD (*n* = 4; two-tailed unpaired Student’s *t* test, ***p value < 0.001); Mr (kDa), relative molecular mass in kilo Daltons (kDa). **b** Renca cells were treated with the indicated concentrations of doxorubicin for 16 h. Western blot shows p53 and HSP90 as loading control. Quantification of signal intensities was accomplished by densitometric analysis and normalization to the respective loading controls. Graph displays mean ± SD (*n* = 2); Mr (kDa), relative molecular mass in kilo Daltons (kDa). **c** Renca cells were treated with the indicated concentrations of MS-275 for 24 h and 48 h. Independent triplicates were analyzed for *p53* mRNA expression by qPCR analysis. Graph shows mean ± SD (*n* = 3; one-way ANOVA; ****p value < 0.0001). **d** Renca cells were treated with the indicated concentrations of MS-275 (μM) and VPA (mM) for 24 h. Expression of p53 was analyzed by Western blot. HSP90 serves as loading control; Mr (kDa), relative molecular mass in kilo Daltons (kDa)

The literature does not provide insights into the impact of the R210C mutation on p53 functions and how it might affect a remaining p53 wild-type allele. Therefore, we experimentally assessed p53 in Renca cells. We compared the p53 expression of Renca cells and the pancreatic primary tumor cell line PPT-5436. Both cell lines are of murine origin, but PPT-5436 cells harbor the characterized point mutation p53^R172H^ that results in a stabilized and transcriptionally inactive p53 (Conradt et al. 2012; Freed-Pastor and Prives 2012; Schneider et al. 2010). We noted that the expression of p53 in PPT-5436 cells was about sixfold higher than in Renca cells (Fig. 2a).

These results prompted us to test for an accumulation of p53 in response to genotoxic stress, which indicates wild-type p53 (Schneider et al. 2010). When we analyzed the p53 expression of Renca cells in response to doxorubicin treatment, we found that total p53 protein expression was upregulated in response to this genotoxic stimulus (Fig. 2b).

It is known that HDACi decrease the expression of wild-type and mutant p53 through a transcriptional mechanism in pancreatic and colorectal cancer cells (Göder et al. 2018; Schäfer et al. 2017; Sonnemann et al. 2014; Stojanovic et al. 2017). When we measured the expression of *p53* mRNA in Renca cells, we detected time- and dose-dependent effects of class I HDACi on *p53* mRNA expression. A treatment of Renca cells with 1.5 µM MS-275 for 48 h led to a significant reduction of *p53* mRNA to 46.5 ± 1.34% of control levels. This effect was more pronounced at higher doses of MS-275 (Fig. 2c).

Immunoblot analyses revealed that this reduction of the *p53* mRNA translated into reduced levels of the p53 protein after 24-h incubations with MS-275 or VPA (Fig. 2d).

These data suggest that HDACi repress the expression of wild-type p53 and p53^R210C^ in Renca cells.

### HDAC inhibition does not promote chemoresistance

Since wild-type p53 is a tumor suppressor (Gottifredi and Wiesmüller 2018; Klusmann et al. 2016), its reduction by HDACi raises concerns that such drugs promote chemoresistance. Furthermore, HDACi-induced alterations in EMT factors (Kiweler et al. 2018) may promote the mesenchymal phenotype that is linked to chemoresistance (Fischer et al. 2015; Zheng et al. 2015). To address these concerns, we incubated Renca cells with combinations of HDACi, and the commonly used chemotherapeutics L-OHP, a DNA crosslinking agent that damages DNA directly, and HU, a ribonucleotide reductase inhibitor that can lead to DNA double-strand breaks secondary to a stalling of replication forks (Nikolova et al. 2017).

**Fig. 3:**
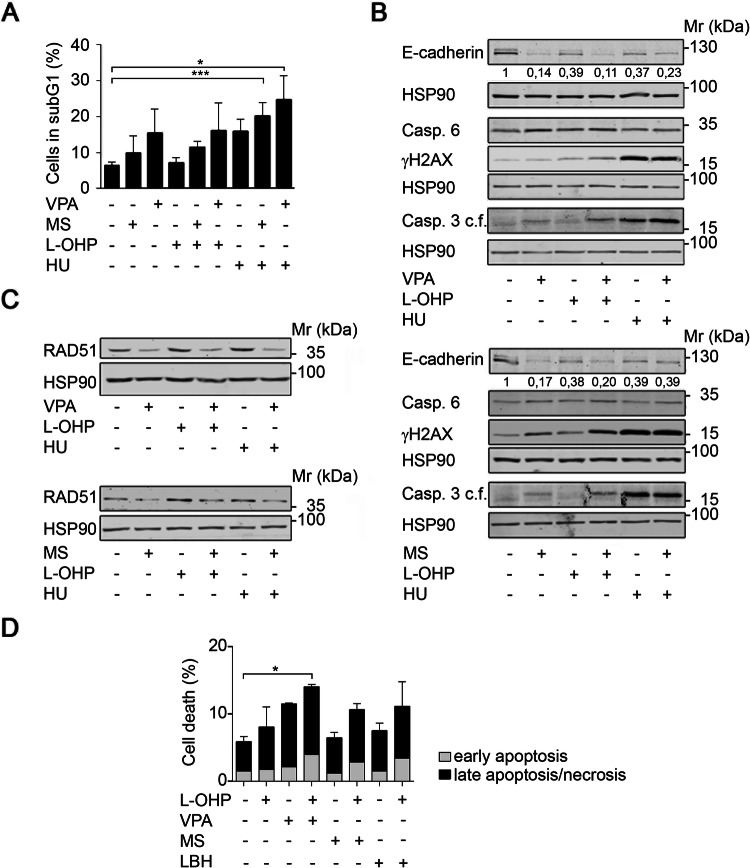
HDACi interact with chemotherapeutics. **a** Renca cells were pre-treated for 24 h with 1.5 mM VPA or 1.5 μM MS-275 and subsequently treated with 5 μM L-OHP or 1 mM HU for 24 h. Cell death was accessed as % subG1 population in fixed, PI-stained cells using flow cytometry. Graph shows mean ± SD (*n* = 3); *p value = 0.0101; ***p value = 0.003; one-way ANOVA. **b** Renca cells were treated with 1.5 mM VPA, 1.5 μM MS-275, 5 μM L-OHP, and 1 mM HU as indicated for 48 h. Expression levels of the indicated proteins were analyzed by Western blot. Band intensities for E-cadherin are indicated, with untreated cells set as 1 (*n* = 2). HSP90 serves as loading control; relative molecular mass in kilo Daltons (kDa). **c** Renca cells were pre-treated for 24 h with 1.5 mM VPA or 1.5 μM MS-275 and subsequently treated with 5 μM L-OHP and 1 mM HU for 24 h. Expression of RAD51 was assessed by Western blot. HSP90 serves as loading control; relative molecular mass in kilo Daltons (kDa) (*n* = 2). **d** H1299-TO-p53 cells were treated with HDACi (2 µM MS-275, 30 nM LBH-589, 3 mM VPA) and/or oxaliplatin (5 µM L-OHP) for 24-48 h. Annexin V/PI-stained cells were subjected to flow cytometry for cell death analyses; *n* = 3 ± SD; *p value < 0.05, two-way ANOVA

Flow cytometric analyses to measure cell death induction showed that Renca cells were resistant to L-OHP and slightly sensitive to HU (Fig. 3a). Such a poor response to chemotherapeutics is a typical feature of RCC (Barbieri et al. 2017; Chang et al. 2019; Piva et al. 2016). Combined treatment of Renca cells with VPA or MS-275 and L-OHP or HU augmented cytotoxic effects of HU significantly (Fig. 3a).

Moreover, the co-treatment with the HDACi and L-OHP or HU reduced the cellular adhesion factor E-cadherin, led to an accumulation of caspase-6, and induced caspase-3 cleavage (Fig. 3b). In addition, MS-275 and VPA slightly increased the accumulation of ɣH2AX that is caused by L-OHP (Fig. 3b) and this was linked to an abrogated induction of RAD51 by L-OHP and HU in Renca cells (Fig. 3c).

To further extend these findings, we analyzed cells derived from another notoriously chemoresistant tumor, non-small cell lung cancer (NSCLC). We incubated human H1299 lung cancer cells with L-OHP, because platinum compounds are a standard therapy for lung tumors (Foy et al. 2017; Gelsomino et al. 2019). In accordance with the data obtained in renal cancer cell lines, three structurally different HDACi (VPA, MS-275, LBH-589) promoted anti-proliferative effects of L-OHP against H1299 cells (Fig. 3d).

We sum up that HDACi do not attenuate drug sensitivity, but rather increase the efficacy of well-established anti-cancer drugs.

### HDACi modulate the expression of adhesion factors

**Fig. 4:**
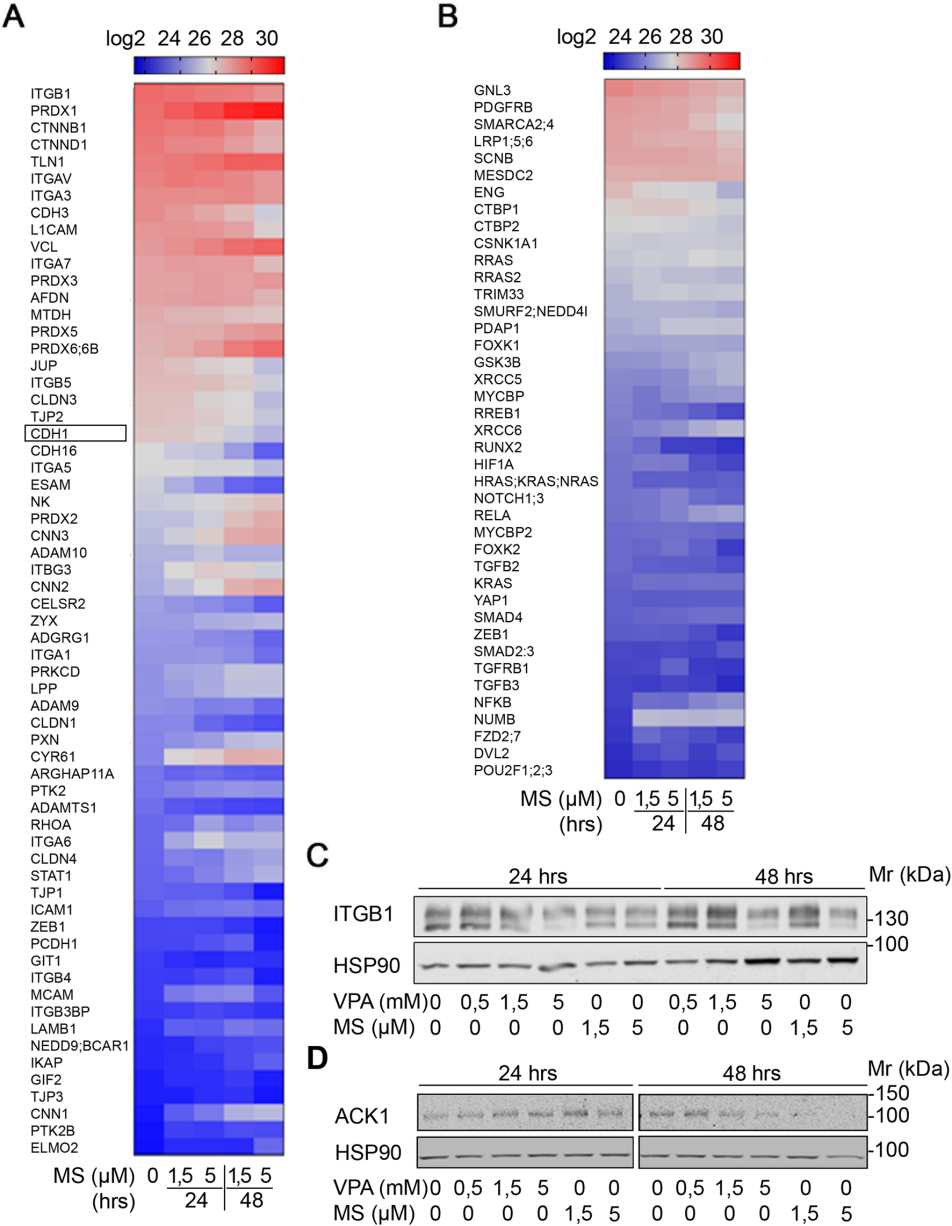
HDACi decrease EMT-associated protein expression. **a, b** Renca cells were treated with the indicated concentrations of MS-275 (μM) for 24–48 h. Four independent replicates were analyzed for protein expression via LFQ in mass spectrometry (**a** adhesion, **b** epithelial/mesenchymal cell identity). Heatmap depicts changes in LFQ expression levels of the indicated proteins. E-cadherin (also termed CDH1) is marked by a frame in **a**. **c** Renca cells were treated with the indicated concentrations of VPA (mM) and MS-275 (μM) for 24 h and 48 h. ITGB1 expression was analyzed by Western blot. HSP90 serves as loading control. **d** Renca cells were treated with the indicated concentrations of VPA (mM) and MS-275 (μM) for 24 h and 48 h. ACK1 expression was analyzed by Western blot. HSP90 serves as loading control

Enhanced chemotherapy resistance and altered cell adhesion and signaling are hallmark features of the metastatic process (Brabletz et al. 2018; Fidler and Kripke 2015; Hao et al. 2019; Nieto 2013; Zeisberg and Neilson 2009). Our analysis of the impact of HDACi on chemosensitivity revealed that class I HDACi in combination with L-OHP and HU attenuated the expression of E-cadherin (Fig. 3b). Therefore, we further analyzed proteome data for the expression of a large number of cell adhesion factors and cytoskeletal proteins. We noted that MS-275 dysregulated a large number of such factors, including E-cadherin [Fig. 4a, b and (Kiweler et al. 2018)].

Proteomics also showed a reduction of integrin-β1 by HDACi (Fig. 4a), which is a key factor for cell adhesion to collagen and the colonization of tumor cells at distant tissues (Chua et al. 2010). Immunoblot analysis verified that 5 µM MS-275 and 5 mM VPA decreased the levels of integrin-β1 (Fig. 4c).

Since overexpression and somatic mutations in the tyrosine kinase ACK1 promote oncogenic signaling, cell proliferation, EMT, and chemoresistance (Chua et al. 2010; Jenkins et al. 2018; Mahajan and Mahajan 2015; Mahajan et al. 2018; Mahendrarajah et al. 2017; Yeh et al. 2012), we additionally tested whether HDACi have an impact on ACK1 in Renca cells. Western blot analyses showed that MS-275 and VPA attenuated ACK1 in a time- and dose-dependent manner (Fig. 4d).

These data demonstrate that HDACi dysregulate proteins that control the metastatic spread to and the colonization of secondary tissues by transformed cells.

### HDACi suppress TGFβ-induced cellular plasticity

Cell adhesion and cellular plasticity are key steps in the progression from a local tumor to a distantly spread metastasis. TGFβ promotes EMT, metastasis, and drug resistance of cancer cells (Brabletz et al. 2018; Fidler and Kripke 2015; Hao et al. 2019; Nieto 2013; Zeisberg and Neilson 2009).

**Fig. 5:**
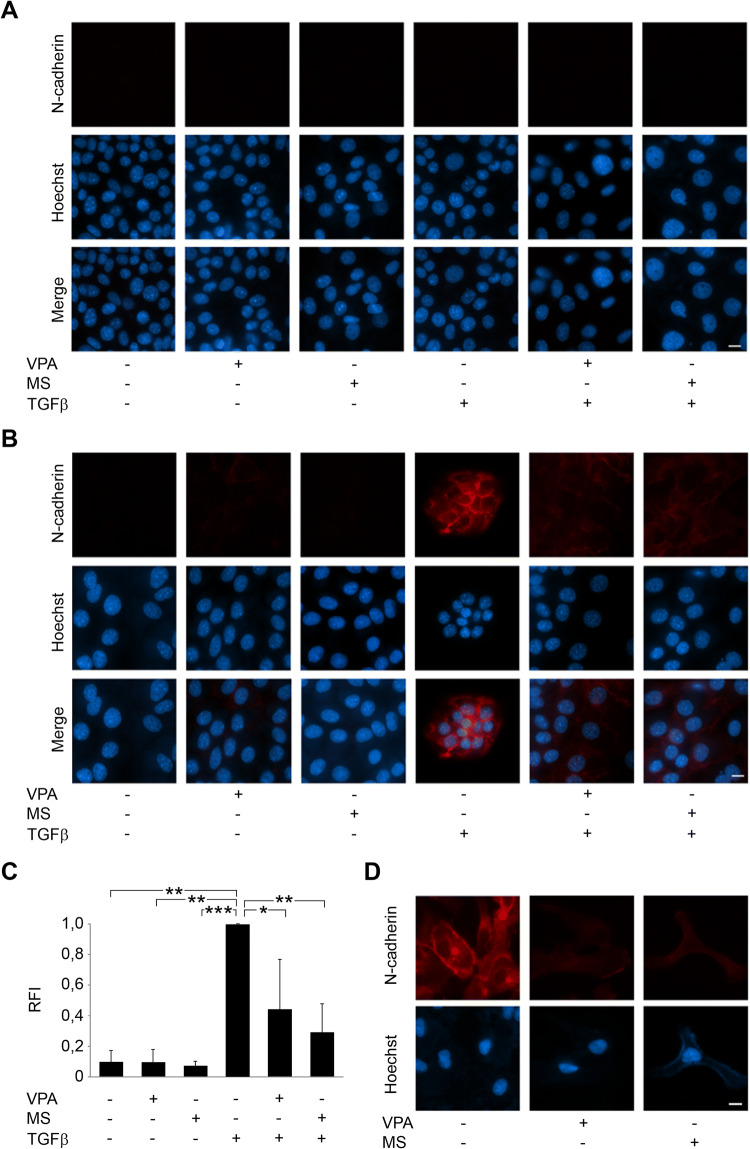
Abrogation of basal and TGFβ-induced expression in transformed cells. **a** Renca cells were treated with HDACi (1.5 mM VPA, 5 µM MS-275), TGFβ (5 ng/ml), and combinations for 48 h. Immunofluorescence shows absence of N-cadherin (α-N-cadherin Ab (BD Bioscience) and α-mouse Cy3 secondary antibody (Dianova)). Nuclei were marked by Hoechst-33258 dye (blue); scale bar, 10 μm. **b** HDACi prevent N-cadherin (red) induction by TGFβ in NM18 cells from mouse mammary gland. **c** Relative fluorescence intensity (RFI) of N-cadherin staining in NM18 cells was determined from three individual experiments. Mean values are shown; *p value < 0.05; **p value < 0.01; ***p value < 0.001; one-way ANOVA. **d** Levels of N-cadherin in primary human RCC cells (Mz-ccRCC2) that were left untreated or exposed to 1.5 mM VPA or 5 µM MS-275

As an upregulation of the cell adhesion molecule N-cadherin is a marker for mesenchymal cells and described to be upregulated in epithelial cells as a consequence of EMT induction after TGFβ treatment (Hao et al. 2019; Zeisberg and Neilson 2009), we analyzed N-cadherin expression in HDACi-treated Renca cells. In immunocytochemistry analyses, immunoblot, and mass-spectrometric proteome analyses, we could not detect any N-cadherin expression in resting and TGFβ-treated Renca cells (Fig. 5a and data not shown).

As a substitute, we analyzed N-cadherin levels in mammary epithelial NM18 cells, which represent a standard TGFβ-sensitive cell system (Deckers et al. 2006). Immunocytochemistry of NM18 cells revealed that VPA and MS-275 significantly reduced TGFβ-induced N-cadherin expression (Fig. 5b, c).

Furthermore, we show in primary Mz-ccRCC2 cells that HDACi abolish a basal expression of N-cadherin (Fig. 5d).

These results suggest that HDACi dysregulate the expression of cell adhesion factors and impair TGFβ-induced cell plasticity.

## Discussion

Our data illustrate that HDACi promote an induction of replicative stress and DNA damage in cultured cancer cells. This finding is coherent with previously published works, which illustrate that HDACs are required for the expression of factors mediating DNA damage, the recognition and repair of DNA lesions, and for scheduled origin firing (Conti et al. 2010; Miller et al. 2010; Nikolova et al. 2017; Noack et al. 2017; Wang et al. 2012; Wells et al. 2013). Our proteomics approach shows that the HDACi-induced accumulation of ɣH2AX in Renca cells is linked to a disturbed expression of proteins that stabilize DNA replication forks and contribute to DNA damage recognition and repair. For example, we demonstrate that VPA and MS-275 diminish the expression levels of RAD51, which is a key HR protein and a survival factor for cancer cells harboring damaged DNA. These findings are consistent with literature evidence from other tumor-derived cells (Göder et al. 2018; Krumm et al. 2016; Nikolova et al. 2017). The observed correlation between DNA damage and prolonged growth arrest of HDACi-treated Renca cells is coherent with DNA damage being a major trigger of cell cycle arrest (Kiweler et al. 2018; Lanz et al. 2019; Nikolova et al. 2017). Nevertheless, more investigations are necessary to assess the relative contribution of replication stress/DNA damage to HDACi-induced anti-proliferative effects. For instance, HDACi-induced alterations of proteins that control apoptosis and autophagy are further pathways through which HDACi might restrict tumor cell growth (Koeneke et al. 2015; Mrakovcic et al. 2019; Vancurova et al. 2018). Moreover, our proteome analyses show HDACi-induced alterations of proteins that control immune tolerance (Kiweler et al. 2018), raising the possibility that HDACi combat tumors through immune modulation.

Despite being a frequently used model system to analyze RCC in vitro and in vivo as syngeneic mouse model (Kiweler et al. 2018), the p53 status of Renca cells was undefined. While wild-type p53 is a short-lived protein, the majority of mutations in p53 are missense mutations that lead to the stable expression a p53 protein variant with a prolonged half-life (Conradt et al. 2012; Freed-Pastor and Prives 2012). We observed a strongly diminished expression of total p53 protein in Renca cells in comparison to a cell line expressing a defined mutant p53 isoform. This lower expression of p53 in Renca cells suggests its wild-type status. Our finding of an accumulation of p53 in doxorubicin treated Renca cells supports this notion. Nonetheless, one allele of p53 carries an ill-defined R210C exchange (Zeitouni et al. 2017). In general, p53 wild-type expression in Renca cells would correspond to the majority of cells derived from common renal cancers. *TP53* mutation rates in this disease are exceptionally low in comparison to other cancer types, with 2.5% for renal papillary-cell carcinoma and 2.4% for renal clear-cell carcinoma (Wang et al. 2017).

Since wild-type p53 can suppress tumorigenesis (Gottifredi and Wiesmüller 2018; Klusmann et al. 2016), the reduction of p53 in HDACi-treated Renca cells appears to be counterintuitive with the anti-proliferative effects of HDACi. However, p53 might not be inactivated and its reduction by HDACi is not complete. There is, for example, an accumulation of p21, which is positively regulated by p53, and a repression of survivin, which is negatively regulated by p53 in HDACi-treated Renca cells (Kiweler et al. 2018). Apparently, the reduction of total p53 may not necessarily lead to a suppression of p53 target gene regulation, because p53 is also activated by acetylation. For example, low and very active levels of acetylated p53 can drive the expression of its target genes and apoptosis upon replication stress and DNA damage in colorectal cancer cells (Brandl et al. 2012). On the other hand, we may also detect p53-independent growth arrest and cell death induction by HDACi in Renca cells, as seen in p53-negative colorectal cancer cells (Sonnemann et al. 2014). Moreover, replication stress triggers apoptosis and mitotic catastrophe after HDACi treatment despite a reduced expression of p53 and its target genes (Göder et al. 2018). One should additionally consider that there are even cases in which p53 antagonizes apoptosis induction (Barckhausen et al. 2014) and the HDACi-evoked loss of various DNA repair proteins including p53 may trigger cytotoxic DNA damage. In conclusion, the observed loss of p53 expression in HDACi-treated Renca cells is not linked to diminished cytotoxic responses or an induction of chemoresistance.

Two recent studies point out that the mesenchymal transition of transformed cells ties in with the resistance of pancreatic and breast cancer cells against DNA-damaging agents (Fischer et al. 2015; Zheng et al. 2015). So far, our data illustrate that HDACi themselves attenuate the expression of DNA repair and promote cell death of HU-treated RCC and NSCLC cultures. Studies including small groups of patients treated with HU and HDACi suggest that such combinatorial treatment might be successful (Bug et al. 2005; Müller and Krämer 2010). Undoubtedly, additional in vivo evidence is necessary to clarify the therapeutic validity of HDACi/HU combination treatment schedules. This also applies to the doses that can be achieved without significant toxicity in RCC patients. A recent report found that up to 1.51 µM MS-275 was achieved without gross toxicity in mice, but large variation of maximal plasma concentrations from 4 to 53.1 ± 92.4 and a half-life from 33.4 to 150 h occurred in humans (Connolly et al. 2017; Kurmasheva et al. 2019), indicating unexplained large patient-to-patient variability. The maximum-tolerated dose of VPA was reported to range, for example, from 50 mg/kg daily to 140 mg/kg/day, which is within the therapeutic serum concentrations of VPA from 0.35 to 0.7 mM (Bug et al. 2005; Münster et al. 2007; Phiel et al. 2001).

In addition to the effects of HDACi on replication stress/DNA damage, their impact on EMT needs to be investigated. Preceding work demonstrated a dysregulation of various proteins that control cell adhesion and migratory properties in RCC cells following class I HDAC inhibition (Kiweler et al. 2018) and we verify an HDACi-mediated downregulation of integrin-β1 in Renca cells. This finding is coherent with the literature that reports an inhibition of integrin-α/β expression and their downstream signaling pathways in HDACi-treated RCC cells (Jones et al. 2009b). However, a plethora of additional factors determines EMT in HDACi-treated cells. For example, we see that HDACi decrease RhoA, which regulates EMT and interacts with HDACs (Mertsch and Krämer 2017). Likewise, proteomics suggests an HDACi-induced increase in the inducible transcription factor STAT1 in Renca cells and we found that such an accumulation of STAT1 contributes to apoptosis in HDACi-treated melanoma cells (Krämer et al. 2006). Another example is ACK1, for which evidence collected in various tumor types suggests a pro-tumorigenic role (Chua et al. 2010; Jenkins et al. 2018; Mahajan and Mahajan 2015; Mahajan et al. 2018). Furthermore, data collected with gastric and liver cancer cells show that ACK1 is overexpressed in primary clinical specimen and that ACK1 promotes the invasive capacity and EMT of such tumor cells through its direct activating effects on AKT kinases (Lei et al. 2015; Xu et al. 2015). AKT also contributes to the spread of RCC cells into bone tissue and a hyperstabilized, mutant ACK1 isoform promotes hallmarks of cancer in RCC cells (Chua et al. 2010). Hence, the reduction of ACK1 by HDACi could cause anti-proliferative, therapeutically relevant effects. We found that HDACi decrease ACK1 by a caspase-dependent mechanism in leukemic cells (Mahendrarajah et al. 2016) and we see that the decline in ACK1 is linked to apoptosis of Renca cells [(Kiweler et al. 2018) and this study]. Accordingly, the loss of ACK1 could occur through caspase-dependent degradation. Additional experiments are necessary to clarify whether ACK1 degradation in renal cancer presents a functional signal for cell growth reduction, cell death induction, and metastasis or poses a downstream marker of cell death.

TGFβ-induced EMT signaling promotes metastasis, chemoresistance, angiogenesis, and immune evasion of tumor cells (Hao et al. 2019). Although the treatment with TGFβ induces N-cadherin in other cell lines (Mikami et al. 2016; Zeisberg and Neilson 2009), Renca cells do not express the TGFβ receptor-II (Engel et al. 1999) and, therefore, fail to accumulate N-cadherin. Such a loss of TGFβ receptor II is also seen in 31 out of 62 RCC patients and correlates with a lower apoptotic index and statistically significant lower survival rates (Miyajima et al. 2003). The expression of E-cadherin, the cytoplasmic localization of β-catenin (Kiweler et al. 2018), and the absence of the mesenchymal marker N-cadherin (this work) verify that Renca cells remain epithelial cells independent of HDACi treatment. In contrast to this, HDACi suppress TGFβ-induced N-cadherin expression in mammary epithelial cells and HDACi decrease basal N-cadherin expression in primary human RCC cells. Our finding that both epithelial (Renca cells) and mesenchymal (Mz-ccRCC2 cells) respond with an induction of cell death to class I HDACi shows that such drugs can kill cells having either differentiation status. Further studies are underway to address if HDACi shift transformed cells to certain molecular signatures of one of the states and thereby eliminate them.

Taken together, our work illustrates that class I HDACi evoke DNA damage and suppress the metastasis-associated phenotype. These data suggest exploiting HDACi further for the treatment of cancer.

## Materials and methods

### Cell culture conditions and drugs

Fetal calf serum (FCS) was from Gibco Invitrogen Life Technologies, Darmstadt, Germany (catalogue numbers 102270/10270106, EU approved origin: South America, lots. 41Q8207K/42G8258K). Renca cells grow at 37 °C and 5% CO_2_ in RPMI medium (Sigma-Aldrich, Munich, Germany) supplemented with 10% FCS, 1% penicillin/streptomycin and 2% glutamine. NM18 and H1299-TO-p53 cells grow at 37 °C and 5% CO_2_ in DMEM medium (Sigma-Aldrich, Munich, Germany) supplemented with 10% FCS and 1% penicillin/streptomycin; 1% glutamine and 5 µg/mL insulin were added to NM18 cells. Renca cells were obtained from Prof. W. Wels, GSH Frankfurt/Main, Germany (derived from a spontaneously developed renal cortical adenocarcinoma in a male Balb/c mouse; ATCC® CRL-2947™). PPT-5436 were developed in the group of one of the coauthors (G.S.). These are a low passage cell line from primary pancreatic tumors of a *PTF1a/p48*^*ex1Cre/*+^*;LSL-KRAS*^G1*2D/*+^*;LSL-p53R172H*^+*/*+^*;LSL-R26*^*Tva−lacZ/−*^ mouse. H1299-TO-p53 were given to us by Dr. G. Rohaly, HPI, Hamburg, Germany. These are a derivative of NCI-H1299 (ATCC® CRL-5803™) cells, which are epithelial cells from a metastatic site lymph node of a lung carcinoma of a male patient. NM18 cells are a subclone of NMuMG cells (ATCC® CRL-1636™), which were isolated by their strong response to TGFβ by Deckers et al. (2006). NMuMG cells are epithelial mammary gland cells from a Namru strain mouse. Mz-ccRCC2 renal tumor cells were isolated as described in Haber et al. (2015) from tumor specimens that were obtained shortly after nephrectomy. Tumor tissue was dissociated mechanically and with 1 mg/ml collagenase II, pressed through a cell strainer (70 μm) and centrifuged under sterile conditions. The obtained cells were first cultured in AmnioMAX C100 Basal Medium including AmnioMAX C100 Supplement (Gibco, Life Technologies, Darmstadt, Germany). After the first passage, they were transferred to DMEM medium supplemented with 10% FCS and 1% penicillin/streptomycin and cultured at 37 °C and 5% CO_2_; i.e., conditions as for Renca cells. The epithelial origin was confirmed by immunohistochemical cytokeratin staining. For the experiments, the cells were used in passages 2–8. No commonly mischaracterized cells were used and cells were tested free of mycoplasma every 4–8 weeks. TGFβ and VPA were purchased from Sigma-Aldrich, Germany. Entinostat was purchased from Selleckchem, Germany.

### Database search for p53 in Renca cells

To search for information on the p53 status of Renca cells, we considered the International Agency for Research on Cancer TP53 Database (IARC, https://p53.iarc.fr/), the Catalogue Of Somatic Mutations In Cancer (COSMIC, https://cancer.sanger.ac.uk/cosmic), the Cancer Cell Line Encyclopedia (CCLE, https://portals.broadinstitute.org/ccle), the American Type Culture Collection (ATCC, https://www.atcc.org*),* the *TP53 website cell line compendium* (https://p53.fr), and Charles River (Zeitouni et al. 2017). Reanalysis of WES data is based on accession no. PRJEB12925 (Mosely et al. 2017).

### Protein lysates, Western blot, densitometric analysis, and antibodies

We have recently summarized the Western blot method used to collect data shown here (Stojanovic et al. 2017). Data acquisition was performed with the Odyssey Infrared Imaging System (Licor), using IRDye® 680RD-coupled or IRDye® 800CW-coupled secondary antibodies. Immunoblots are representative of at least two independent experiments. The following antibodies were used: Cell Signaling Technology (Frankfurt/Main, Germany): cleaved caspase-3/#9661, caspase-6/#9762, E-cadherin/#3195; Enzo Life Sciences (Lörrach, Germany): HSP90/ADI-SPA-830-F; Merck Millipore (Darmstadt, Germany): AB1952; Santa Cruz Biotechnology (Heidelberg, Germany): ACK1/sc-28336, pS139-H2AX/sc-101696 (ɣH2AX), β-actin/sc-47778, P53/sc81168; Abcam: RAD51/ab63801; BD Bioscience (Heidelberg, Germany): N-cadherin/BD610921.

### Cell cycle and cell death analysis

Cells were harvested with trypsin/EDTA and fixed with 80% ethanol. Samples were then stored at − 20 °C for at least 2 h. Thereafter, cells were incubated with RNAse A (Carl Roth; final concentration 20 µg/mL) for 1 h at RT and stained with propidium iodide (PI) (Sigma-Aldrich; final concentration 16.5 µg/mL) for 10 min on ice. Annexin V/PI staining was performed according to the manufacturer’s instructions with Annexin V-FITC (Becton Dickinson) and PI at RT for 15 min in the dark. Cells were subjected to flow cytometry with the FACSCanto Flow Cytometer (BD Biosciences). Data were analyzed with the FACSDIVA™ Software (BD Biosciences).

### Immunofluorescence to detect N-cadherin

NM18 cells were seeded on chamber coverslips (µ-Slide 8-well, iBidi) and treated with 1.5 mM VPA or 5 µM MS-275, 5 ng/mL TGFβ, or combinations thereof for 48 h. Cells were washed thrice with phosphate-buffered saline (PBS), and fixed for 20 min in 4% paraformaldehyde. After cell permeabilization using 0.1% Triton X-100 in PBS for 10 min, cells were stained for 1 h at RT using α-N-cadherin antibody (BD Bioscience) 1:100 in PBS + 1% FCS. After incubation, slides were washed three times for 5 min in PBS and incubated for 1 h at RT with α-mouse Cy3 secondary antibody (Dianova). DNA/cell nuclei were visualized by staining with 0.5 µg/mL Hoechst-33258 (Sigma-Aldrich). Observation, image acquisition, and analysis of stained cells were performed using AxioVert 200 M, a digital AxioCam CCD camera and Axiovision software (Carl Zeiss, Jena, Germany).

### Proteomics and pathway analysis

NuPAGE® LDS Sample Buffer (1×) supplemented with 100 µM DTT was added to treated cells and controls. Lysates were subjected to mass spectrometry (Dejung et al. 2016) for protein detection. Proteins were run on gels and digested with mass spectrometry-grade trypsin (Sigma-Aldrich, St. Louis, Missouri) and purified with a StageTip. Data were analyzed with Maxquant v1.5.2.8 using LFQ, ENSEMBL GRCm38 peptide database (57,751 entries), and custom R scripts. (Dejung et al. 2016). Full proteomics data are available upon scientific request. Preceding statistical analysis by R software version 3.2 using unpaired *t* test (two conditions) or one-way ANOVA (multiple conditions) resulted in an individual set of significantly regulated proteins. The background set was composed of all proteins successfully quantified in the experiment (5812 proteins). For each set of significantly regulated proteins, three hypergeometric tests (for biological processes, for molecular functions, and for cellular components) were performed using the R package “GOstats”. By this means, it was determined if the GO terms that were associated with significantly changing genes were over-represented over the defined background (Falcon and Gentleman 2007). For each protein listed, the Entrez gene ID was obtained using the annotation R package “BiomaRt”; see also cells (Kiweler et al. 2018).

### Determination of mRNA transcripts

The primer sequences that we used to determine p53 transcript numbers were *AGAGACCGCCGTACAGAAGA* (forward)/*CTGTAGCATGGGCATCCTTT* (reverse); β-actin: *GTCGAGTCGCGTCCACC* (forward)/*GTCATCCATGGCGAACTGGT* (reverse). Isolation of total RNA was carried out by means of the RNeasy Mini-Kit (Qiagen, Hilden, Germany). To quantify the relative gene expression by ∆∆-Ct method, 20 ng cDNA and 100 nM primer mix (MWG Biotech, Ebersberg, Germany) were mixed together with the Power SybrGreen master mix and applied to the StepOnePlus real-time PCR device (Applied Biosystems/Thermo Fischer, Frankfurt/Main, Germany). Primer efficiencies were previously determined by the formula 10^(−1/slope)^. All experiments were carried out in technical and biological triplicates.

### Statistical analysis

One-way ANOVA/two-way ANOVA were used as indicated for the respective experiments (GraphPad Prism Vers.6.01) and corrected for multiple testing using Dunnett’s test or Bonferroni’s multiple comparisons test. Two-paired *t* test with Welch’s correction was used when not more than two conditions were compared and for proteomics as comparison with untreated samples. *p* values are indicated in the legends.

## Electronic supplementary material

Below is the link to the electronic supplementary material.

Supplementary Fig. S1 Protein levels and statistical evaluation by two-tailed unpaired t-test with Welch’s correction, **** p-value < 0.0001, *** p-value < 0.001, ** p-value < 0.01, * p-value < 0.05 (PDF 45 kb)

Supplementary Fig. S2 WES data (accession no. PRJEB12925) for Renca cells from (Mosely et al. 2017) demonstrating a monoallelic mutation in p53 expressed in Renca cell (PNG 234 kb)

## References

[CR1] Aiello NM, Brabletz T, Kang Y et al (2017) Upholding a role for EMT in pancreatic cancer metastasis. Nature 547:E7–E8. 10.1038/nature22963 28682339 10.1038/nature22963 PMC5830071

[CR2] Barbieri CE, Chinnaiyan AM, Lerner SP et al (2017) The emergence of precision urologic oncology: a collaborative review on biomarker-driven therapeutics. Eur Urol 71:237–246. 10.1016/j.eururo.2016.08.024 27567210 10.1016/j.eururo.2016.08.024 PMC5195855

[CR3] Barckhausen C, Roos WP, Naumann SC, Kaina B (2014) Malignant melanoma cells acquire resistance to DNA interstrand cross-linking chemotherapeutics by p53-triggered upregulation of DDB2/XPC-mediated DNA repair. Oncogene 33:1964–1974. 10.1038/onc.2013.141 23604128 10.1038/onc.2013.141

[CR4] Bayat Mokhtari R, Homayouni TS, Baluch N et al (2017) Combination therapy in combating cancer. Oncotarget 8:38022–38043. 10.18632/oncotarget.16723 28410237 10.18632/oncotarget.16723 PMC5514969

[CR5] Brabletz T, Kalluri R, Nieto MA, Weinberg RA (2018) EMT in cancer. Nature reviews. Cancer 18:128–134. 10.1038/nrc.2017.118 29326430 10.1038/nrc.2017.118

[CR6] Bradner JE, Mak R, Tanguturi SK et al (2010) Chemical genetic strategy identifies histone deacetylase 1 (HDAC1) and HDAC2 as therapeutic targets in sickle cell disease. Proc Natl Acad Sci USA 107:12617–12622. 10.1073/pnas.1006774107 20616024 10.1073/pnas.1006774107 PMC2906581

[CR7] Brandl A, Wagner T, Uhlig KM et al (2012) Dynamically regulated sumoylation of HDAC2 controls p53 deacetylation and restricts apoptosis following genotoxic stress. J Mol Cell Biol 4:284–293. 10.1093/jmcb/mjs013 22493095 10.1093/jmcb/mjs013

[CR8] Bug G, Ritter M, Wassmann B et al (2005) Clinical trial of valproic acid and all-trans retinoic acid in patients with poor-risk acute myeloid leukemia. Cancer 104:2717–2725. 10.1002/cncr.21589 16294345 10.1002/cncr.21589

[CR9] Chang AJ, Zhao L, Zhu Z et al (2019) The past, present and future of immunotherapy for metastatic renal cell carcinoma. Anticancer Res 39:2683–2687. 10.21873/anticanres.13393 31177102 10.21873/anticanres.13393

[CR10] Chua BT, Lim SJ, Tham SC et al (2010) Somatic mutation in the ACK1 ubiquitin association domain enhances oncogenic signaling through EGFR regulation in renal cancer derived cells. Mol Oncol 4:323–334. 10.1016/j.molonc.2010.03.001 20359967 10.1016/j.molonc.2010.03.001 PMC5527913

[CR11] Chun P (2018) Therapeutic effects of histone deacetylase inhibitors on kidney disease. Arch Pharmacal Res 41:162–183. 10.1007/s12272-017-0998-7 10.1007/s12272-017-0998-7 29230688

[CR12] Connolly RM, Rudek MA, Piekarz R (2017) Entinostat: a promising treatment option for patients with advanced breast cancer. Future Oncol 13:1137–1148. 10.2217/fon-2016-0526 28326839 10.2217/fon-2016-0526 PMC5618943

[CR13] Conradt L, Henrich A, Wirth M et al (2012) Mdm2 inhibitors synergize with topoisomerase II inhibitors to induce p53-independent pancreatic cancer cell death. Int J Cancer. 10.1002/ijc.27916 23115126 10.1002/ijc.27916

[CR14] Conti C, Leo E, Eichler GS et al (2010) Inhibition of histone deacetylase in cancer cells slows down replication forks, activates dormant origins, and induces DNA damage. Cancer Res 70:4470–4480. 10.1158/0008-5472.CAN-09-3028 20460513 10.1158/0008-5472.CAN-09-3028 PMC2880188

[CR15] Deckers M, van Dinther M, Buijs J et al (2006) The tumor suppressor Smad4 is required for transforming growth factor beta-induced epithelial to mesenchymal transition and bone metastasis of breast cancer cells. Cancer Res 66:2202–2209. 10.1158/0008-5472.CAN-05-3560 16489022 10.1158/0008-5472.CAN-05-3560

[CR16] Dejung M, Subota I, Bucerius F et al (2016) Quantitative proteomics uncovers novel factors involved in developmental differentiation of *Trypanosoma brucei*. PLoS Pathog 12:e1005439. 10.1371/journal.ppat.1005439 26910529 10.1371/journal.ppat.1005439 PMC4765897

[CR17] Dietrich MF, Gerber DE (2016) Chemotherapy for advanced non-small cell lung cancer. Cancer Treat Res 170:119–149. 10.1007/978-3-319-40389-2_6 27535392 10.1007/978-3-319-40389-2_6

[CR18] Engel JD, Kundu SD, Yang T et al (1999) Transforming growth factor-beta type II receptor confers tumor suppressor activity in murine renal carcinoma (renca) cells. Urology 54:164–170 10414746 10.1016/s0090-4295(99)00093-x

[CR19] Falcon S, Gentleman R (2007) Using GOstats to test gene lists for GO term association. Bioinformatics 23:257–258. 10.1093/bioinformatics/btl567 17098774 10.1093/bioinformatics/btl567

[CR20] Fidler IJ, Kripke ML (2015) The challenge of targeting metastasis. Cancer Metastasis Rev 34:635–641. 10.1007/s10555-015-9586-9 26328524 10.1007/s10555-015-9586-9 PMC4661188

[CR21] Fischer KR, Durrans A, Lee S et al (2015) Epithelial-to-mesenchymal transition is not required for lung metastasis but contributes to chemoresistance. Nature 527:472–476. 10.1038/nature15748 26560033 10.1038/nature15748 PMC4662610

[CR22] Foy V, Schenk MW, Baker K et al (2017) Targeting DNA damage in SCLC. Lung cancer 114:12–22. 10.1016/j.lungcan.2017.10.006 29173760 10.1016/j.lungcan.2017.10.006

[CR23] Freed-Pastor WA, Prives C (2012) Mutant p53: one name, many proteins. Genes Dev 26:1268–1286. 10.1101/gad.190678.112 22713868 10.1101/gad.190678.112 PMC3387655

[CR24] Gelsomino F, Lamberti G, Parisi C et al (2019) The evolving landscape of immunotherapy in small-cell lung cancer: a focus on predictive biomarkers. Cancer Treat Rev 79:101887. 10.1016/j.ctrv.2019.08.003 31491661 10.1016/j.ctrv.2019.08.003

[CR25] Göder A, Emmerich C, Nikolova T et al (2018) HDAC1 and HDAC2 integrate checkpoint kinase phosphorylation and cell fate through the phosphatase-2A subunit PR130. Nat Commun 9:764. 10.1038/s41467-018-03096-0 29472538 10.1038/s41467-018-03096-0 PMC5823910

[CR26] Gottifredi V, Wiesmüller L (2018) The tip of an iceberg: replication-associated functions of the tumor suppressor p53. Cancers. 10.3390/cancers10080250 30060597 10.3390/cancers10080250 PMC6115784

[CR27] Haber T, Jöckel E, Roos FC et al (2015) Bone metastasis in renal cell carcinoma is preprogrammed in the primary tumor and caused by AKT and integrin alpha5 signaling. J Urol 194:539–546. 10.1016/j.juro.2015.01.079 25623744 10.1016/j.juro.2015.01.079

[CR28] Hao Y, Baker D, Ten Dijke P (2019) TGF-beta-mediated epithelial-mesenchymal transition and cancer metastasis. Int J Mol Sci. 10.3390/ijms20112767 31195692 10.3390/ijms20112767 PMC6600375

[CR29] Jenkins C, Luty SB, Maxson JE et al (2018) Synthetic lethality of TNK2 inhibition in PTPN11-mutant leukemia. Sci Signal. 10.1126/scisignal.aao5617 30018082 10.1126/scisignal.aao5617 PMC6168748

[CR30] Jones J, Juengel E, Mickuckyte A et al (2009a) The histone deacetylase inhibitor valproic acid alters growth properties of renal cell carcinoma in vitro and in vivo. J Cell Mol Med 13:2376–2385. 10.1111/j.1582-4934.2008.00436.x 18657224 10.1111/j.1582-4934.2008.00436.x PMC6512366

[CR31] Jones J, Juengel E, Mickuckyte A et al (2009b) Valproic acid blocks adhesion of renal cell carcinoma cells to endothelium and extracellular matrix. J Cell Mol Med 13:2342–2352. 10.1111/j.1582-4934.2008.00603.x 19067765 10.1111/j.1582-4934.2008.00603.x PMC6529979

[CR32] Juengel E, Nowaz S, Makarevi J et al (2014) HDAC-inhibition counteracts everolimus resistance in renal cell carcinoma in vitro by diminishing cdk2 and cyclin A. Mol Cancer 13:152. 10.1186/1476-4598-13-152 24935000 10.1186/1476-4598-13-152 PMC4073177

[CR33] Kiweler N, Brill B, Wirth M et al (2018) The histone deacetylases HDAC1 and HDAC2 are required for the growth and survival of renal carcinoma cells. Arch Toxicol 92:2227–2243. 10.1007/s00204-018-2229-5 29845424 10.1007/s00204-018-2229-5

[CR34] Klusmann I, Rodewald S, Müller L et al (2016) p53 activity results in DNA replication fork processivity. Cell Rep 17:1845–1857. 10.1016/j.celrep.2016.10.036 27829155 10.1016/j.celrep.2016.10.036

[CR35] Koeneke E, Witt O, Oehme I (2015) HDAC family members intertwined in the regulation of autophagy: a druggable vulnerability in aggressive tumor entities. Cells 4:135–168. 10.3390/cells4020135 25915736 10.3390/cells4020135 PMC4493453

[CR36] Krämer OH, Baus D, Knauer SK, Stein S, Jäger E, Stauber RH, Grez M, Pfitzner E, Heinzel T (2006) Acetylation of Stat1 modulates NF-kappaB activity. Genes Dev 20(4):473–485 16481475 10.1101/gad.364306 PMC1369049

[CR37] Krumm A, Barckhausen C, Kucuk P et al (2016) Enhanced histone deacetylase activity in malignant melanoma provokes RAD51 and FANCD2-triggered drug resistance. Cancer Res 76:3067–3077. 10.1158/0008-5472.CAN-15-2680 26980768 10.1158/0008-5472.CAN-15-2680

[CR38] Kurmasheva RT, Bandyopadhyay A, Favours E et al (2019) Evaluation of entinostat alone and in combination with standard-of-care cytotoxic agents against rhabdomyosarcoma xenograft models. Pediatr Blood Cancer 66:e27820. 10.1002/pbc.27820 31099166 10.1002/pbc.27820 PMC6685061

[CR39] Lanz MC, Dibitetto D, Smolka MB (2019) DNA damage kinase signaling: checkpoint and repair at 30 years. EMBO J 38:e101801. 10.15252/embj.2019101801 31393028 10.15252/embj.2019101801 PMC6745504

[CR40] Lei X, Li YF, Chen GD et al (2015) Ack1 overexpression promotes metastasis and indicates poor prognosis of hepatocellular carcinoma. Oncotarget 6:40622–40641. 10.18632/oncotarget.5872 26536663 10.18632/oncotarget.5872 PMC4747357

[CR41] Mahajan K, Mahajan NP (2015) ACK1/TNK2 tyrosine kinase: molecular signaling and evolving role in cancers. Oncogene 34:4162–4167. 10.1038/onc.2014.350 25347744 10.1038/onc.2014.350 PMC4411206

[CR42] Mahajan NP, Coppola D, Kim J et al (2018) Blockade of ACK1/TNK2 to squelch the survival of prostate cancer stem-like cells. Sci Rep 8:1954. 10.1038/s41598-018-20172-z 29386546 10.1038/s41598-018-20172-z PMC5792546

[CR43] Mahendrarajah N, Paulus R, Krämer OH (2016) Histone deacetylase inhibitors induce proteolysis of activated CDC42-associated kinase-1 in leukemic cells. J Cancer Res Clin Oncol 142:2263–2273. 10.1007/s00432-016-2229-x 27576506 10.1007/s00432-016-2229-x PMC11819266

[CR44] Mahendrarajah N, Borisova ME, Reichardt S et al (2017) HSP90 is necessary for the ACK1-dependent phosphorylation of STAT1 and STAT3. Cell Signal 39:9–17. 10.1016/j.cellsig.2017.07.014 28739485 10.1016/j.cellsig.2017.07.014

[CR45] Mao S, Lu G, Lan X et al (2017) Valproic acid inhibits epithelial–mesenchymal transition in renal cell carcinoma by decreasing SMAD4 expression. Mol Med Rep 16:6190–6199. 10.3892/mmr.2017.7394 28901475 10.3892/mmr.2017.7394

[CR46] Mertsch S, Krämer OH (2017) The interplay between histone deacetylases and rho kinases is important for cancer and neurodegeneration. Cytokine Growth Factor Rev 37:29–45. 10.1016/j.cytogfr.2017.05.006 28606734 10.1016/j.cytogfr.2017.05.006

[CR47] Mikami S, Oya M, Mizuno R et al (2016) Recent advances in renal cell carcinoma from a pathological point of view. Pathol Int 66:481–490. 10.1111/pin.12433 27461942 10.1111/pin.12433

[CR48] Miller KM, Tjeertes JV, Coates J et al (2010) Human HDAC1 and HDAC2 function in the DNA-damage response to promote DNA nonhomologous end-joining. Nat Struct Mol Biol 17:1144–1151. 10.1038/nsmb.1899 20802485 10.1038/nsmb.1899 PMC3018776

[CR49] Miyajima A, Asano T, Seta K et al (2003) Loss of expression of transforming growth factor-beta receptor as a prognostic factor in patients with renal cell carcinoma. Urology 61:1072–1077. 10.1016/s0090-4295(02)02553-0 12736050 10.1016/s0090-4295(02)02553-0

[CR50] Mosely SI, Prime JE, Sainson RC et al (2017) Rational selection of syngeneic preclinical tumor models for immunotherapeutic drug discovery. Cancer Immunol Res 5:29–41. 10.1158/2326-6066.CIR-16-0114 27923825 10.1158/2326-6066.CIR-16-0114

[CR51] Mrakovcic M, Kleinheinz J, Fröhlich LF (2019) p53 at the crossroads between different types of HDAC inhibitor-mediated cancer cell death. Int J Mol Sci. 10.3390/ijms20102415 31096697 10.3390/ijms20102415 PMC6567317

[CR52] Müller S, Krämer OH (2010) Inhibitors of HDACs–effective drugs against cancer? Curr Cancer Drug Targets 10:210–228 20201785 10.2174/156800910791054149

[CR53] Münster P, Marchion D, Bicaku E et al (2007) Phase I trial of histone deacetylase inhibition by valproic acid followed by the topoisomerase II inhibitor epirubicin in advanced solid tumors: a clinical and translational study. J Clin Oncol 25:1979–1985. 10.1200/JCO.2006.08.6165 17513804 10.1200/JCO.2006.08.6165

[CR54] Nieto MA (2013) Epithelial plasticity: a common theme in embryonic and cancer cells. Science 342:1234850. 10.1126/science.1234850 24202173 10.1126/science.1234850

[CR55] Nikolova T, Kiweler N, Krämer OH (2017) Interstrand crosslink repair as a target for HDAC inhibition. Trends Pharmacol Sci 38:822–836. 10.1016/j.tips.2017.05.009 28687272 10.1016/j.tips.2017.05.009

[CR56] Noack K, Mahendrarajah N, Hennig D et al (2017) Analysis of the interplay between all-trans retinoic acid and histone deacetylase inhibitors in leukemic cells. Arch Toxicol 91:2191–2208. 10.1007/s00204-016-1878-5 27807597 10.1007/s00204-016-1878-5 PMC6063353

[CR57] Phiel CJ, Zhang F, Huang EY et al (2001) Histone deacetylase is a direct target of valproic acid, a potent anticonvulsant, mood stabilizer, and teratogen. J Biol Chem 276:36734–36741. 10.1074/jbc.M101287200 11473107 10.1074/jbc.M101287200

[CR58] Piva F, Giulietti M, Santoni M et al (2016) Epithelial to mesenchymal transition in renal cell carcinoma: implications for cancer therapy. Mol Diagn Therapy 20:111–117. 10.1007/s40291-016-0192-5 10.1007/s40291-016-0192-5 26940073

[CR59] Rogakou EP, Pilch DR, Orr AH et al (1998) DNA double-stranded breaks induce histone H2AX phosphorylation on serine 139. J Biol Chem 273:5858–5868. 10.1074/jbc.273.10.5858 9488723 10.1074/jbc.273.10.5858

[CR60] Schäfer C, Göder A, Beyer M et al (2017) Class I histone deacetylases regulate p53/NF-kappaB crosstalk in cancer cells. Cell Signal 29:218–225. 10.1016/j.cellsig.2016.11.002 27838375 10.1016/j.cellsig.2016.11.002

[CR61] Schneider G, Henrich A, Greiner G et al (2010) Cross talk between stimulated NF-kappaB and the tumor suppressor p53. Oncogene 29:2795–2806. 10.1038/onc.2010.46 20190799 10.1038/onc.2010.46

[CR62] Singla M, Kumar A, Bal A et al (2018) Epithelial to mesenchymal transition induces stem cell like phenotype in renal cell carcinoma cells. Cancer Cell Int 18:57. 10.1186/s12935-018-0555-6 29681769 10.1186/s12935-018-0555-6 PMC5896088

[CR63] Sonnemann J, Marx C, Becker S et al (2014) p53-dependent and p53-independent anticancer effects of different histone deacetylase inhibitors. Br J Cancer 110:656–667. 10.1038/bjc.2013.742 24281001 10.1038/bjc.2013.742 PMC3915118

[CR64] Stojanovic N, Hassan Z, Wirth M et al (2017) HDAC1 and HDAC2 integrate the expression of p53 mutants in pancreatic cancer. Oncogene 36:1804–1815. 10.1038/onc.2016.344 27721407 10.1038/onc.2016.344

[CR65] Tretbar S, Krausbeck P, Müller A et al (2019) TGF-beta inducible epithelial-to-mesenchymal transition in renal cell carcinoma. Oncotarget 10:1507–1524. 10.18632/oncotarget.26682 30863498 10.18632/oncotarget.26682 PMC6407676

[CR66] Vancurova I, Uddin MM, Zou Y, Vancura A (2018) Combination therapies targeting HDAC and IKK in solid tumors. Trends Pharmacol Sci 39:295–306. 10.1016/j.tips.2017.11.008 29233541 10.1016/j.tips.2017.11.008 PMC5818305

[CR67] Wang H, Zhou W, Zheng Z et al (2012) The HDAC inhibitor depsipeptide transactivates the p53/p21 pathway by inducing DNA damage. DNA Repair 11:146–156. 10.1016/j.dnarep.2011.10.014 22112863 10.1016/j.dnarep.2011.10.014

[CR68] Wang Z, Peng S, Jiang N et al (2017) Prognostic and clinicopathological value of p53 expression in renal cell carcinoma: a meta-analysis. Oncotarget 8:102361–102370. 10.18632/oncotarget.21971 29254251 10.18632/oncotarget.21971 PMC5731961

[CR69] Wei M, Mao S, Lu G et al (2018) Valproic acid sensitizes metformin-resistant human renal cell carcinoma cells by upregulating H3 acetylation and EMT reversal. BMC Cancer 18:434. 10.1186/s12885-018-4344-3 29665787 10.1186/s12885-018-4344-3 PMC5902941

[CR70] Wells CE, Bhaskara S, Stengel KR et al (2013) Inhibition of histone deacetylase 3 causes replication stress in cutaneous T cell lymphoma. PLoS ONE 8:e68915. 10.1371/journal.pone.0068915 23894374 10.1371/journal.pone.0068915 PMC3718806

[CR71] Xu SH, Huang JZ, Xu ML et al (2015) ACK1 promotes gastric cancer epithelial-mesenchymal transition and metastasis through AKT-POU2F1-ECD signalling. J Pathol 236:175–185. 10.1002/path.4515 25678401 10.1002/path.4515

[CR72] Ye X, Brabletz T, Kang Y et al (2017) Upholding a role for EMT in breast cancer metastasis. Nature 547:E1–E3. 10.1038/nature22816 28682326 10.1038/nature22816 PMC6283276

[CR73] Yeh YC, Lin HH, Tang MJ (2012) A tale of two collagen receptors, integrin beta1 and discoidin domain receptor 1, in epithelial cell differentiation American journal of physiology. Cell Physiol 303:C1207–1217. 10.1152/ajpcell.00253.2012 10.1152/ajpcell.00253.2012 23015544

[CR74] Yoshikawa M, Hishikawa K, Marumo T, Fujita T (2007) Inhibition of histone deacetylase activity suppresses epithelial-to-mesenchymal transition induced by TGF-beta1 in human renal epithelial cells. J Am Soc Nephrol 18(1):58–65 17135397 10.1681/ASN.2005111187

[CR75] Zeisberg M, Neilson EG (2009) Biomarkers for epithelial-mesenchymal transitions. J Clin Investig 119:1429–1437. 10.1172/JCI36183 19487819 10.1172/JCI36183 PMC2689132

[CR76] Zeitouni B, Tschuch C, Davis JM et al (2017) Abstract 1840: whole-exome somatic mutation analysis of mouse cancer models and implications for preclinical immunomodulatory drug development. Cancer Res 77(13 Suppl):Abstract nr 1840. doi: 10.1158/1538-7445.AM2017-1840

[CR77] Zheng X, Carstens JL, Kim J et al (2015) Epithelial-to-mesenchymal transition is dispensable for metastasis but induces chemoresistance in pancreatic cancer. Nature 527:525–530. 10.1038/nature16064 26560028 10.1038/nature16064 PMC4849281

