# Supplementary figures and images for: Histone deacetylase inhibitors dysregulate DNA repair proteins and antagonize metastasis-associated processes
Source: J Cancer Res Clin Oncol. 2020 Jan 13;146(2):343–56. doi: 10.1007/s00432-019-03118-4 (PMC6985217; doi:10.1007/s00432-019-03118-4)

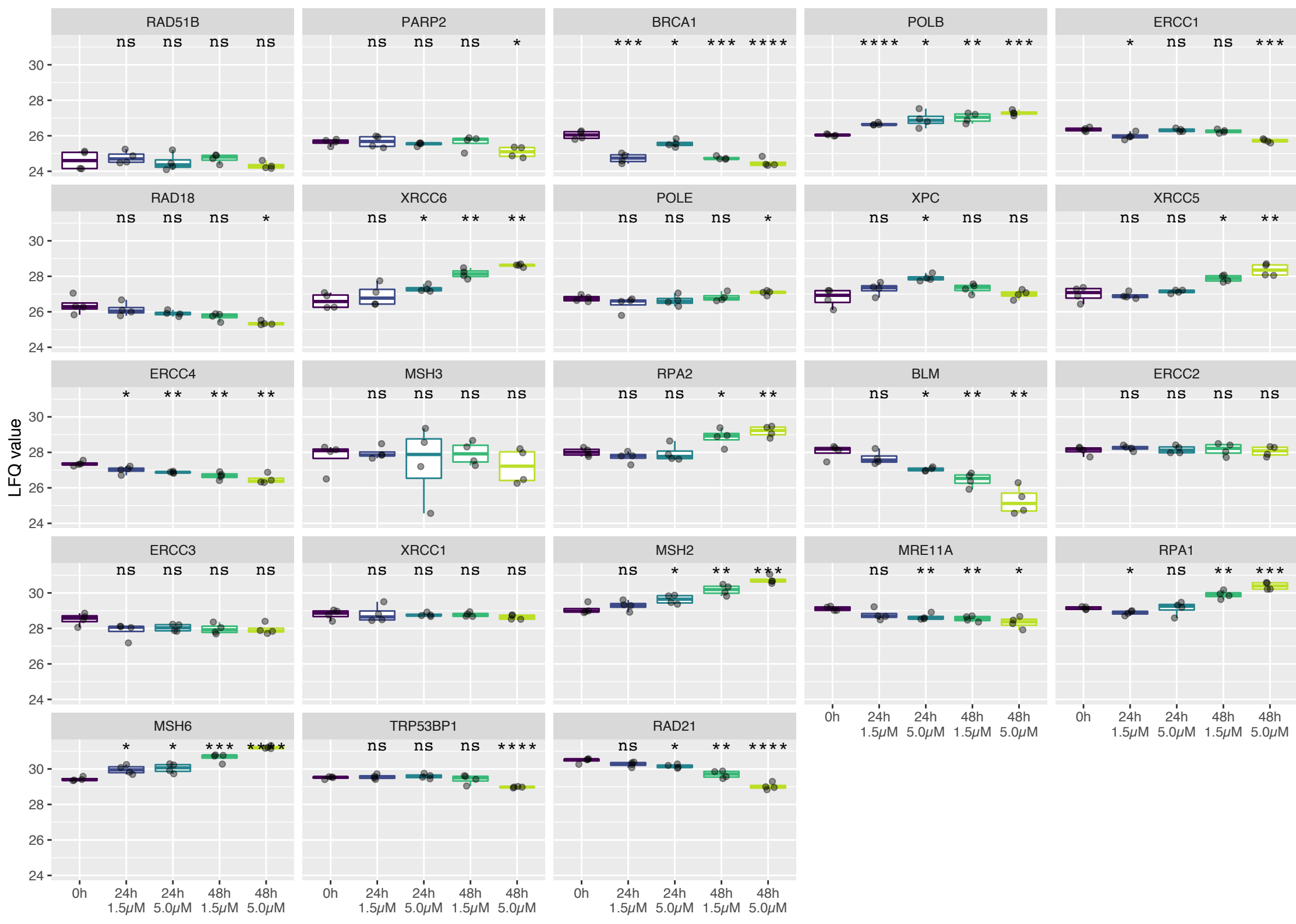

Supplement: Supplementary file 1 — Supplementary Fig. S1 Protein levels and statistical evaluation by two-tailed unpaired t-test with Welch’s correction, **** p-value < 0.0001, *** p-value < 0.001, ** p-value < 0.01, * p-value < 0.05 (PDF 45 kb) [file 432_2019_3118_MOESM1_ESM.pdf]

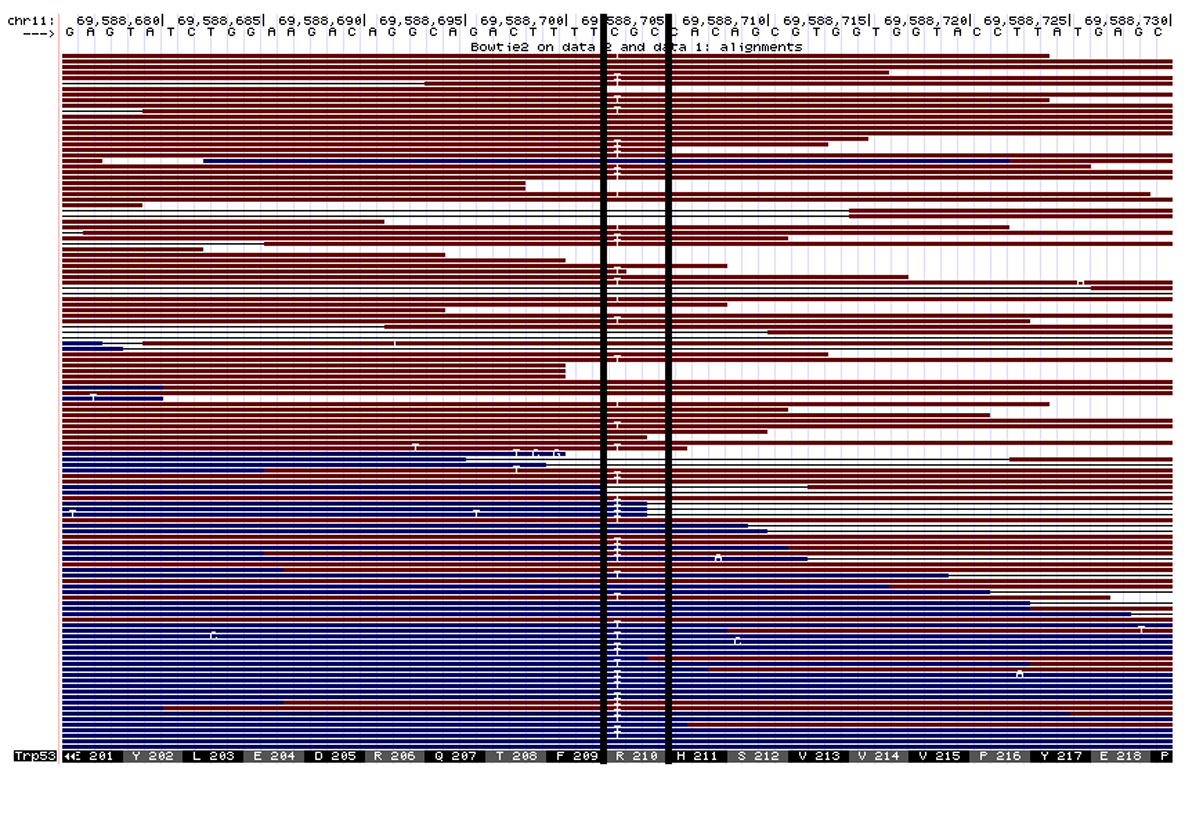

Supplement: Supplementary file 2 — Supplementary Fig. S2 WES data (accession no. PRJEB12925) for Renca cells from (Mosely et al. 2017) demonstrating a monoallelic mutation in p53 expressed in Renca cell (PNG 234 kb) [file 432_2019_3118_MOESM2_ESM.png]
